# Professional Fidelity

**Published:** 1871-07

**Authors:** L. Parmele


### PROFESSIONAL FIDELITY.

BY DR. L. PARMELE.

**Read before the Connecticut State Dental Association.**

"A capital subject," said a gentleman the other day, since our programme was announced, "but I think there will be little said about it." Whether this indicates the moral status of the dental profession, is a matter of which I will express no opinion. It would *seem*, however, to indicate the *impression* of the one who made the remark. As this is the topic upon which we are to exchange thoughts, I will proceed.

There are many ways to take money from a man's pocket, sometimes the process is summary and concise, at others it is wily and insidious. If you are met on the highway by a ruffian who demands your money or your life, you call him a robber. If a man stealthily enters your premises and takes away your goods, you call him a thief. If by false representations the size of your pocket book is diminished you pronounce it a swindle, and so on to the end. But for each of these you have a legal remedy, *provided* only you are fortunate enough to catch the *scoundrel.*

There are other modes, however, of obtaining money, scarcely less *reprehensible* or *dishonest,* where redress is not so easy. The petty tricks and deceptions resorted to whereby money may be obtained without rendering an equivalent are so common, that a man almost expects to be cheated at every turn, unless he places himself in the attitude of continual watchfulness, and one is nearly driven to the conclusion that dishonesty is the rule and honesty the exception in his dealing with the world.

But I need not enlarge. Your own imaginations will readily complete the picture. It may be set in a frame at your leisure, and might with advantage be hung up at the corner of every street, for the unwary to look upon and take warning. The familiar words so often seen at our railroad depots, "Beware of Pickpockets," might perhaps, appropriately be added.

I think it may be safely laid down as a principle, that whoever voluntarily assumes any duty where the interest and well being of others is involved, should by every principle of honesty, so to fit and qualify himself for his calling, that those who shall place their confidence in him may not suffer at his hands. This responsiblity can in no way be set aside.

There is perhaps no occupation in life which requires, and really demands so various and peculiar qualities and qualifications, as those needed to make up the character of an efficient and successful dentist.

To render himself agreeable and acceptable to his patrons, and secure their confidence, he must be frank, affable and courteous. To preserve equanimity of temper under all provocations,—and there will be many,—he must possess calmness and patience. He must have habits of neatness; his manipulations must be gentle, his deportment kind and forbearing, and to grapple successfully with all the duties devolving upon him, he must be at once mechanic, surgeon, dentist and physician. These, tempered with discretion and judgment, are some of the leading requisites to success in the practice of dentistry. And yet, while each and all of these may be possessed in a high degree, if he be deficient in that principle or quality which forms the first subject for our consideration, he lacks the one thing above all others which should recommend him to public confidence and patronage, and so far as his real character is known and read of men, he will neither have the one nor deserve the other. As the mariner's compass is the only reliance by which he guides his craft over the pathless sea, so fidelity is the only principle of action which renders a man careful and exact in the performance of obligations devolving upon him.

In years gone by, when the means of instruction and information were meager, and men were compelled to depend upon their own ingenuity and what little they could spy out with a sharp eye, a degree of ignorance might be winked at somewhat; but now it is far otherwise. With the light of science and experience radiating from so many sources; with opportunities and advantages so numerous, there can be no apology for entering upon the practice of dentistry without those qualifications which will enable one to discharge the important trust, with propriety and skillfulness.

A man has no right thus either to dishonor his calling, or to impose upon the community. If he does so, he does it in defiance of the principle of common honesty, and in violation of the golden rule. Surely such an one can lay no claim to serving his patrons with fidelity.

Should it be objected that such a course of preparation as here indicated, would be expensive, and consume too much time, I would suggest that the shorter method be adopted of "drawing at sight."

We have a just right to expect fidelity in the tradesman, the mechanic, the farmer, the domestic servant and in our friend, and whoever fails to perform his part in accordance with either stipulated or an implied understanding becomes, in some sense at least, a swindler—he is obtaining goods under false pretenses.

The man, who, having spent a few weeks with some one who perhaps knew little more of dentistry than himself, puts out his sign with "Surgeon Dentist" in glittering letters, followed by an advertisement in some public print, accompanied with a flattering editorial—probably written by himself,—setting forth his extraordinary merits, generously paying the editor a handsome fee to endorse the lie, becomes no less a swindler than the man who sells you chalk for cheese, or calcined plaster for wheat flour, and as richly merits the condemnation of the community. Nay, more so, for in the one case you discover the cheat in time to save your stomach, and you only lose your money. In the other you lose your money, but the cheat is not disclosed until too late, when your teeth have followed it.

This is no overdrawn picture gotten up for exhibition. It has in it more truth than poetry.

Do you ask then if all advertising should be discountenanced? I suppose a man has an undoubted right to speak the truth. Where, when, and how he shall do it, becomes a matter of expediency and discretion with him. If he sees fit to speak it through the common newspaper, the privilege is his, only let him "speak the truth and lie not."

What is fidelity? Defined by Webster, it is "faithfulness, careful and exact observance of duty or performance of obligation." What then does fidelity demand of the dentist, if he is called upon for advice? It demands that his examinations shall be *unselfish*, *careful* and *thorough*, and that advice be given accordingly. That he shall *not* in order to secure an engagement, so express himself as to raise expectations not likely to be realized, and which will probably or possibly result, not only in disappointment to the patient, but in discredit to himself and to the profession.

In his deportment towards, and in relation to other dentists, it requires that he should be honorable and friendly, always avoiding the use of language designed to create unjust and false impressions, or to cast undeserved reproach upon their character or reputation.

It does not, however, require that their crimes or misdemeanors shall be concealed.

There is, I regret to say, a habit among dentists of hurrying through their operations with little regard to the quality or character, as if the money, and the shortest possible time in which it could be grasped, were the only things to be thought of or cared for, which deserves severe censure. Such services are not worth the material wasted. They are sure to fail—have brought much discredit on our profession, and victimized many an unfortunate. I speak not of the price charged, but of the injustice—the cheat. In filling a tooth the object desired is its preservation. If for want of thoroughness it results in failure, the patient has not received what he bargained for. He is victimized—swindled. A man must put his own estimate upon his services. When that is done, let them be rendered with fidelity.

Fidelity also requires, not only that a man shall render strictly and always equivalent service for pecuniary consideration, but it takes him further. There are obligations reaching deeper, higher, and beyond all considerations of mere dollars and cents. There are duties which man owes to his fellow man from which even the dentist is not exempt, and which demand *sometimes* service without compensation, except so far as it brings its own reward, and which often outweighs all pecuniary consideration. There will be instances in the practice of every dentist wherein he will be called to exercise his liberality and benevolence.

If we see a man suffering with hunger, is it not our duty to feed him? If he is beset by ruffians, and lies bleeding by the way, shall we not bind up his wounds and provide for his necessities? So, too, in the practice of our profession, we shall often find opportunities and *demands* for the exercise of that charity which "seeketh not her own" and "never faileth."

No man, however, is required to engage in any arduous employment without being entitled to that reward which comes of industry. And it is in justice due to him who shall spend his time and money in properly qualifying himself to be especially useful to others, that he should realize such advantages from his labor as shall place him beyond the reach of want in his advanced years. But the satisfaction arising from a consciousness of having performed well an incumbent duty—of relieving the suffering and unfortunate, as well as of rendering a service equal to the recompence, makes duty to become a pleasure, and sweetens every toil.

So then let it be ours to emulate each other in raising high the standard of our vocation. Let fidelity in living characters be inscribed upon it, and the golden rule as a shining wreath ennoble and adorn it.

